# Genome Sequences of Four Italian *Streptococcus thermophilus* Strains of Dairy Origin

**DOI:** 10.1128/genomeA.00126-14

**Published:** 2014-03-13

**Authors:** Laura Treu, Veronica Vendramin, Barbara Bovo, Stefano Campanaro, Viviana Corich, Alessio Giacomini

**Affiliations:** a Department of Environmental Engineering, Technical University of Denmark, Kongens Lyngby, Denmark; b Department of Agronomy, Food, Natural Resources, Animals and Environment (DAFNAE), University of Padova, Padua, Italy; c Department of Biology, University of Padova, Padua, Italy

## Abstract

This report describes the genome sequences of four *Streptococcus thermophilus* strains, namely, TH982, TH985, TH1477, and 1F8CT, isolated from different dairy environments from the Campania and the Veneto regions in Italy. These data are aimed at increasing the genomic information available on this species, which is of paramount importance for the dairy industry.

## GENOME ANNOUNCEMENT

*Streptococcus thermophilus* is a species of great importance for the preparation of numerous dairy products (1). In fact, besides lactic acid production, several other technological characteristics are possessed by strains of this species.

The first two strains of *S. thermophilus*, TH982 and TH985, were isolated in the Campania region from whey and curd, respectively, obtained from mozzarella di bufala campana DOP cheese (pasta filata cheese) production. *S. thermophilus* strains TH1477 and 1F8CT were isolated in the Veneto region from cow milk and curd, respectively, from Grana Padano DOP cheese (long-ripening cheese) production. These strains possess a variety of functional properties of technological interest for the dairy industry, such as slow (1F8CT) or rapid (TH1477, TH982, and TH985) acidifying capabilities, low fermentation and growth rates (1F8CT), and pigment biosynthesis (TH1477).

Here, we present the genome sequences of *S. thermophilus* strains TH982, TH985, TH1477, and 1F8CT, generated using an Illumina MiSeq platform (with 1-kb mate-pair libraries) at the Ramaciotti Centre, Sydney, Australia. Sequencing coverages of 218×, 183×, 142×, and 261× were obtained corresponding to 1,635,998, 1,410,685, 1,258,918, and 1,544,110 paired-end reads, respectively (2 × 250 bp). The files generated were assembled with Velvet version 1.2.10 (2) and ABySS software version 1.3.5 (3) (optimal *k*-mer, 131). The consensus sequences of the two assemblies were manually compared. Between 52 and 84 scaffolds were obtained, with total sizes of 1,733,024, 1,838,250, 1,879,471, and 1,742,121 bp for strains TH982, TH985, TH1477, and 1F8CT, respectively, with a G+C content of 39%. All strains had the scaffolds assembled into a single circular chromosome by aligning them against the reference genome of *S. thermophilus* CNRZ1066 (assembly ASM1184v1). Several plasmid sequences were detected by BLAST analysis in two scaffolds of strain TH985, in three of TH1477, and in five of 1F8CT.

Protein-coding open reading frames (ORFs) were predicted and annotated using the RAST server (4). The numbers of predicted protein-coding genes (CDSs) in TH982, TH985, TH1477, and 1F8CT strains were 1,924, 1,952, 1,986 and 1,864, respectively. Furthermore, 47, 69, 55, and 51 RNA genes were found, respectively. These genomes contain few phage sequences and no transposase-coding genes, while 20 to 26 clusters of regularly interspaced short palindromic repeats (CRISPRs) were found.

A comparison with the reference genome of *S. thermophilus* CNRZ1066 highlighted numerous CDSs that were exclusive to each strain. In particular, strains from the Veneto region include numerous sequences belonging to the oxidative stress responding genes, while those from Campania contain unique genes for choline and betaine uptake and betaine biosynthesis, which are important in the osmotic stress response.

These data are intended to increase the availability of genomes of *S. thermophilus* strains of dairy origin (5, 6) in order to better understand their biodiversity and their known and potential technological properties.

### Nucleotide sequence accession numbers.

The sequences of this whole-genome shotgun project have been deposited at DDBJ/EMBL/GenBank under the accession no. AZTL00000000, AZTM00000000, AZTJ00000000, and AZTK00000000 for *S. thermophilus* strains TH982, TH985, TH1477, and 1F8CT, respectively. The versions described in this paper are AZTL01000000, AZTM01000000, AZTJ01000000 and AZTK01000000, respectively.
